# Why cells need iron: a compendium of iron utilisation

**DOI:** 10.1016/j.tem.2024.04.015

**Published:** 2024-12

**Authors:** Megan R. Teh, Andrew E. Armitage, Hal Drakesmith

**Affiliations:** 1 MRC Translational Immune Discovery Unit, MRC Weatherall Institute of Molecular Medicine, Radcliffe Department of Medicine, University of Oxford, Oxford, UK

**Keywords:** iron, enzymes, metabolism, mitochondria, bioenergetics

## Abstract

Iron deficiency is globally prevalent, causing an array of developmental, haematological, immunological, neurological, and cardiometabolic impairments, and is associated with symptoms ranging from chronic fatigue to hair loss. Within cells, iron is utilised in a variety of ways by hundreds of different proteins. Here, we review links between molecular activities regulated by iron and the pathophysiological effects of iron deficiency. We identify specific enzyme groups, biochemical pathways, cellular functions, and cell lineages that are particularly iron dependent. We provide examples of how iron deprivation influences multiple key systems and tissues, including immunity, hormone synthesis, and cholesterol metabolism. We propose that greater mechanistic understanding of how cellular iron influences physiological processes may lead to new therapeutic opportunities across a range of diseases.

## Highlights

Iron has a key role in multiple important cellular processes, including gene regulation, metabolism, bioenergetics, and hormone synthesis.
Iron deficiency is globally widespread and associated with several and diverse impacts on health, the underlying mechanisms of which are poorly understood.
We describe potential molecular causative links between cellular iron deficiency and a range of symptoms, and indicate important unknowns in the field.

## Iron is essential for life

Life relies on metal cofactors, including iron, zinc, manganese, copper, and molybdenum [1,2]. Iron specifically is utilised by almost all species, except *Borrelia burgdorferi* and lactobacilli [3., 4., 5.], with roles ranging from structural to enzymatic [6]. Biological systems have co-opted iron due to its ability to transiently bind gaseous ligands and to efficiently catalyse reduction–oxidation (redox) reactions necessary for cellular function [7].

Within mammals, ∼70% of iron is incorporated into red blood cell (RBC) haemoglobin for oxygen transport to tissues [7,8]. Consequently, a common manifestation of iron deficiency is anaemia, with >1.2 billion cases worldwide [9]. Non-anaemic iron deficiency prevalence is even higher [9]. While iron deficiency anaemia classically presents with hypochromic and microcytic RBCs [10], iron deficiency *per se* likely has broader impacts across body systems since all mammalian cells require iron. Absolute iron deficiency results from insufficient dietary iron intake, impaired iron absorption, or blood loss, while functional iron deficiency occurs during inappropriate prolonged tissue iron sequestration, as during chronic inflammation [10]. Iron depletion can also occur locally within tissue niches, such as the tumour microenvironment [11]. Iron deficiency is increasingly recognised to result in multiple pathophysiological effects, including impaired vaccine responses, hair loss, increased cardiac disease risk, and developmental delay [10,12].

Here, we provide a comprehensive overview of the roles of iron in biochemistry, cell biology, and physiology. Where such information is available, we describe how iron deficiency influences these processes, making, in some cases, a first attempt to link underlying molecular mechanisms to diverse iron-related disease manifestations. We then pose new research questions to further translate our growing basic knowledge of iron biology into medical relevance.

## Cellular iron uptake, storage, and trafficking

The beneficial properties of iron for redox reactions also render iron toxic when in excess, generating reactive oxygen species (ROS) via Fenton reactions [13]. The universality of iron usage across kingdoms of life makes it critical for the growth of many pathogens during infection [14]. As a result, mammalian iron homeostasis is tightly regulated, primarily by the hepcidin–ferroportin interaction [7], reviewed extensively elsewhere [15,16]. Here, we describe cellular iron transport and then focus on the many roles of iron in cellular biology.

Cellular iron uptake is canonically mediated by the transferrin receptor (TFRC, TfR1) [17]. TFRC binds iron-bound transferrin, inducing clathrin-mediated endocytosis [17]. Endosomal acidification releases iron from transferrin, and subsequent reduction of Fe^3+^ to Fe^2+^ enables transport into the cytosol, predominantly via DMT1 [NRAMP2/SLC11A2, a **solute carrier (SLC) protein**; see Glossary] [17,18]. Enterocytic DMT1 also mediates direct uptake of dietary non-haem iron [19]. Non-transferrin-bound iron (NTBI) can enter cells via the zinc transporters ZIP8 (SLC39A8) and ZIP14 (SLC39A14) [20,21], while iron bound to the extracellular matrix polysaccharide, hyaluronan, is proposed to bind CD44, with the resulting complex being taken up by endocytosis [22].

Following cellular entry, iron can be mobilised to mitochondria for haem and iron sulfur (Fe-S) cluster biosynthesis, coordinated as an ion via amino acid side-chains into proteins, or stored by ferritin [23]. Ferritin is a 24-subunit cage-like protein complex comprising heavy (FTH) and light (FTL1/2) chain subunits [7], which can store up to ∼4300 iron atoms [24]. Ferritin buffers cellular iron by storing excess iron during abundance and releasing it during scarcity [23]. Iron release is mediated by ferritin breakdown (ferritinophagy) following delivery to autophagosomes by NCOA4 [25]. Trafficking of iron to ferritin and other cellular iron-binding proteins requires metallochaperones, such as PCBP1 and PCBP2 [26]. Fe-S clusters and haem groups are similarly chaperoned by proteins/complexes, such as **glutaredoxin (GLRX)-**3–BOLA2 and PGMRC2, respectively [26,27].

Cellular iron export relies predominantly on ferroportin (SLC40A1) [28., 29., 30., 31.], although iron egress via secretion of ferritin-bound iron in extracellular vesicles and haem export via FLVCR1a, ABCG2, and ABCB5 have also been reported [15,32., 33., 34.].

## Intracellular iron regulation

### Iron regulatory proteins

Intracellular iron is tightly regulated because both iron deficiency and overload are associated with detrimental effects. Classically, intracellular iron is regulated by the iron regulatory protein (IRP)/iron response element (IRE) system [23]. The IRPs, cytosolic aconitase 1 (ACO1, IRP1) and IREB2 (IRP2), are activated by low cellular iron acting post transcriptionally by stabilising transcripts of proteins involved in iron uptake and preventing translation of transcripts encoding iron egress and storage proteins [23]. During iron repletion, ACO1 binds an [4Fe-4S] cluster, enabling catalysis but obstructing its RNA-binding site [23]. Upon cellular iron deficiency, the Fe-S cluster is lost, permitting ACO1 RNA binding [23]. By contrast, IREB2 is degraded during iron repletion following ubiquitination by the [2Fe-2S] cluster-stabilised ubiquitin-ligase, FBXL5 [35,36]. IRPs operate by binding IREs, which are mRNA hairpin loops in the 3′ untranslated regions (UTR) of mRNAs encoding proteins that promote iron uptake (TFRC and DMT1) and in the 5′ UTR of mRNAs linked to iron egress (ferroportin) and storage (FTL1/2, FTH) [23]. During iron deficiency, IRP binding to *TFRC* and *DMT1* 3′-IREs stabilises mRNA by blocking RNase entry, promoting iron uptake, while IRP binding to 5′-IRE-containing mRNAs excludes ribosome binding and translation, suppressing iron egress and storage by ferroportin and ferritin, respectively [23]. During iron scarcity, the IRE-IRP system also downregulates proteins associated with iron usage [37], including the haem synthesis protein, **5-aminolevulinic acid synthase (ALAS)-2** [38], and the tricarboxylic acid (TCA) cycle enzyme, mitochondrial aconitase 2 (ACO2), which requires a [2Fe-2S] cluster for catalysis [39]. Reducing iron use by haem synthesis and the TCA cycle may enable more efficient iron budgeting toward other essential pathways.

### Hypoxia inducible factor

Hypoxia-inducible factor (HIF) proteins are key regulators of the hypoxia response [40]. Under normoxic or iron-replete conditions, HIFα proteins are hydroxylated by PHD enzymes, which are members of a large iron, oxygen, and 2-oxoglutarate [2-OG, α-ketoglutarate (α-KG)]-dependent enzyme family [**2-OG dependent dioxygenases (2-OGDDs)**] [40]. Hydroxylation promotes HIFα ubiquitination by VHL proteins, marking HIFα proteins for degradation [40]. Conversely, when oxygen or iron is limiting, PHD protein inactivity allows HIFα stabilisation and transcriptional activity [40]. HIFα proteins have broad transcriptional activity, but notably regulate metabolism [41]. HIF1α promotes a metabolic shift away from oxygen consumption by upregulating glycolysis and suppressing oxidative phosphorylation (OXPHOS) and fatty acid β-oxidation (both iron-requiring processes, see following section) [41]. Iron chelators and cobalt ions (which displace iron, thereby inactivating PHDs and stabilising HIF1α) have historically been used to study hypoxia responses [42,43].

### Haem-regulated inhibitor

Cellular iron homeostasis is also regulated by haem in erythroid precursors via haem-regulated inhibitor (HRI, EIF2AK1) [44]. During haem repletion, haem binding inhibits the kinase activity of HRI [44]. Conversely, during haem deficiency, HRI becomes active, phosphorylating itself and the translational initiation factor, eIF-2α [44]. eIF-2α phosphorylation blocks globin translation, preventing detrimental accumulation of misfolded globins in the absence of haem [44]. Concurrently, eIF-2α phosphorylation promotes the integrated stress response to support cell survival during haem insufficiency [44]. Recent work identified non-erythroid roles of HRI in the responses to mitochondrial stress and cytosolic protein misfolding [45., 46., 47.]. Whether HRI also has a role in haem sensing in non-erythroid cells remains an open question.

### Tristetraprolin and mTOR

Tristetraprolin (TTP) was recently described to mobilise iron to essential pathways during extended iron depletion by inhibiting nonessential but particularly iron-demanding processes [48., 49., 50.]. During iron limitation, TTP is upregulated and binds to AU-rich elements of mRNA 3′-UTRs, promoting decay of mRNAs encoding **lipoic acid synthase (LIAS)**, ACO2, **NADH:ubiquinone oxidoreductase core subunit S1 (NDUFS1),** and **ubiquinol-cytochrome c reductase, Rieske iron-sulfur polypeptide 1 (UQCRFS1)**, all of which support mitochondrial OXPHOS [48]. Notably, this suggests OXPHOS to be ‘nonessential’ due to the capacity of cells to switch to glycolysis for energy generation [48., 49., 50.]. mTOR, a crucial integrator of diverse metabolic cues, including amino acid depletion, is proposed to act upstream of TTP [49]. mTOR inhibition during iron depletion was recently reported to act via iron sensing by the 2-OGDD protein, **histone lysine demethylase (KDM)-**3B [51]. KDM3B uses iron to catalyse the removal of the repressive histone mark, histone 3 lysine 9 dimethylation (H3K9me2) [51]. Under iron deficiency, KDM3B becomes defective, allowing H3K9me2 accumulation and preventing transcription of the gene encoding the leucine transporter, LAT3 [51]. LAT3 downregulation prevents leucine uptake, inhibiting mTOR via the NPRL2-leucine sensing pathway [51]. mTOR suppression via this pathway may induce TTP.

Therefore, cellular iron is tightly regulated at multiple nodes, involving different forms of iron: Fe-S clusters (IRE-IRP), iron ions (PHD-HIFα and KDM3B-mTOR-TTP) and haem (HRI). Using diverse regulators may facilitate more nuanced responses to specific environmental stimuli. For instance the IRE-IRP system enables rapid responses to acute changes in cellular iron levels by increasing uptake and releasing iron stores [23]. The TTP system is instead proposed to enable cells to survive extended periods of iron scarcity [48., 49., 50.]. How the four axes of cellular iron regulation described (IRE-IRP, PHD-HIF, KDM3B-mTOR-TTP, and haem-HRI) act in concert, and in different cell types across different anatomical sites and tissues where local concentrations of iron may fluctuate substantially, is unknown. Furthermore, iron fluctuations could be detected and responded to by alternative, as yet undiscovered, pathways.

## Iron in cellular biochemistry

Within human cells, iron is predicted to interact with ∼400 different proteins [6]. Iron-interacting proteins broadly utilise iron for its capacity to easily exchange electrons and to form bonds with many other elements in different orientations [7]. Iron may bind proteins as iron ions or complexed into Fe-S clusters or haem groups [6]. These proteins operate in and regulate diverse pathways, which we describe in the following sections, beginning with aspects of gene regulation and DNA metabolism.

## Iron in DNA replication and repair

**Figure 1:**
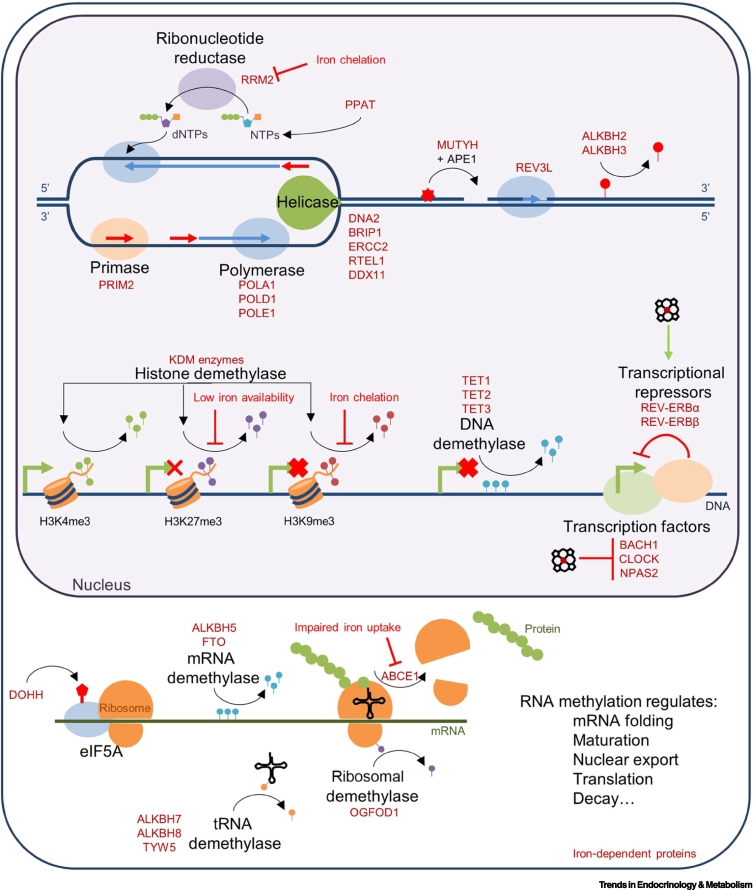
Iron in DNA synthesis, repair, and gene expression. DNA synthesis begins with the unwinding of the DNA helix by helicases, including iron-dependent DNA2. Primase utilises its iron catalytic unit, PRIM2, to deposit a primer, and iron-dependent polymerases elongate the DNA strand. dNTPs necessary for DNA synthesis are produced by iron-dependent ribonucleotide reductase via conversion of rNTPs to dNTPs. Purine synthesis requires the activity of the iron sulfur (Fe-S) cluster-binding initiating enzyme, phosphoribosyl pyrophosphate aminotransferase (PPAT). DNA repair similarly utilises not only iron-dependent helicases, but also iron-dependent MUTYH in base excision repair and alkylation B homolog 2/3 (ALKBH2/3) for repairing alkylation damage. Demethylation of histones, DNA, mRNAs, tRNAs, and the ribosome is mediated by iron-dependent 2-oxoglutarate dependent dioxygenases (2-OGDDs), with crucial roles in maintaining chromatin structure and translational efficiency. The transcription factors BACH1, CLOCK and NPAS2 and the transcriptional repressors REV-ERBα/β are regulated by haem. Iron-dependent deoxyhypusine monooxygenase (DOHH) deposits hypusine on eIF5A to support translational efficiency, while ABCE1 allows for ribosomal recycling. Iron-dependent proteins are denoted in dark red.

DNA replication begins with DNA helix unwinding by helicases, including iron-dependent DNA2 [52] (Figure 1). The Fe-S cluster of DNA2 configures DNA2 for efficient DNA binding and helicase activity [52]. After DNA unwinding, a short RNA primer is deposited for DNA replication initiation [53]. Primase, which comprises two subunits, including iron-dependent PRIM2, places this primer [53]. DNA polymerases Polα, Polβ, and Polε then synthesise the complementary DNA strand using their [4Fe-4S] cluster-binding catalytic subunits (POLA1, POLD1, and POLE1, respectively) [54] and purine and pyrimidine dNTPs. Notably, **phosphoribosyl pyrophosphate aminotransferase (PPAT)**, the initiating enzyme in purine nucleotide synthesis, contains an Fe-S cluster [55]. Meanwhile, RRM2, a component of ribonucleotide reductase (RNR), which converts NTPs to dNTPs, requires an iron core for catalysis [56,57]. Accordingly, iron chelation potently suppresses cancer cell line expansion *in vitro*, in part by impairing RNR [58]. Similarly, low iron availability suppresses primary T cell clonal expansion and cell cycle progression [59]. However, whether iron chelation or deficiency inhibits other components of the DNA replication machinery beyond RNR is unexplored. Virally encoded enzymes important for viral replication, such as Herpes simplex virus RNR and severe acute respiratory syndrome-coronavirus 2 (SARS-CoV-2) helicase and RNA-dependent RNA polymerase, are similarly iron dependent [60., 61., 62.], suggesting that iron starvation may inhibit the replication of certain viruses [63].

Several DNA repair enzymes are iron dependent [54]. Similar to DNA2, the iron-dependent helicases BRIP1 (FANCJ), ERCC2 (XPD), RTEL1, and DDX11 (CHLR1) utilise Fe-S clusters in DNA unwinding, enabling removal of detrimental DNA secondary structures, such as G-quadruplexes, and entry to damaged DNA sites [52,54,64., 65., 66., 67.] (Figure 1). RTEL1 is also critical for telomere maintenance, with mutations resulting in telomere shortening [67]. DNA glycosylases, including iron-dependent MUTYH, mediate initial base removal in base excision repair alongside APE1 [54,68]. Translesional DNA synthesis is then completed by Polζ, which, similar to the DNA replication polymerases, contains an iron-dependent subunit, REV3L [54,69]. DNA bases featuring alkylation damage can be corrected directly by the iron-dependent 2-OGDD family **alkylation B homolog (ALKBH) enzymes**, ALKBH2 and ALKBH3 [70]. These both use iron to catalyse oxidation of aberrant alkyl groups, which spontaneously dissociate as formaldehyde, returning the base to its undamaged state [70].

DNA replication is critical for cellular division, and maintenance of genomic stability is necessary to avoid malignancy. While iron deprivation is known to impair cellular replication, its impact on genome fidelity and mutation remains an open question. This may have particular relevance for tumours, in which regulatory control of proliferation is lost, but where microenvironments may be low in iron due to both local cellular iron acquisition [11] and underlying systemic iron deficiency, which is associated with some cancers [71]. Under these conditions, low iron availability might influence mutagenesis.

## Iron in gene expression

Cell type and function is fundamentally dictated by gene expression, which is regulated at various levels. Iron-dependent enzymes operate at stages including epigenetic regulation, transcription, RNA processing, and translation.

### Iron in epigenetic regulation

Access of the transcriptional machinery to DNA is determined by the chromatin landscape [72], which is modulated by chemical modifications on DNA itself and on histone DNA-packaging proteins [72]. Methylation represents a critical epigenetic marker on DNA and histone substrates [73] (Figure 1). DNA and histone methylation is regulated by methyltransferases, which deposit methyl groups, and demethylases, which remove them [73]. Many DNA and histone demethylases are iron-dependent 2-OGDD enzymes, which hydroxylate methyl groups, producing unstable methyl–hydroxy intermediates that spontaneously dissociate [73]. DNA methylation at cytosine bases, arbitrated by the iron-dependent **Ten-eleven translocases (TETs),** represses gene expression by sterically hindering transcription factor binding and, via recruitment of transcriptional repressors [73,74] and iron chelation, impairs TET2 demethylation activity in *in vitro* adipocytes [75].

Meanwhile, effects of histone methylation depend on where modification occurs. H3K4me3 is an activating histone mark typically located around transcription start sites [72]. H3K9me3 typically denotes transcriptionally silenced regions of the genome, while H3K27me3 represses transcription [72]. Iron chelation impairs the activity of isolated KDM4A [76], and abrogates KDM3A/B demethylation in cells *in vitro* [51,75,77]. KDM3B inhibition results in accumulation of repressive H3K9me2 and decreases transcription of genes encoding leucine importers [51] and cyclins critical for cell cycle progression [77]. Low iron also promotes accumulation of repressive H3K27me3, which is normally removed by KDM6A/B during T helper (Th)-17 CD4+ T cell activation [78]. The sensitivity of DNA and histone demethylation to iron availability suggests a mechanism through which iron controls cell fate and function. Availability of the other 2-OGDD enzyme co-factors, α-KG and oxygen, which require iron in the TCA cycle and haemoglobin, respectively, could also link iron deficiency to histone demethylase activity and epigenetic regulation.

### Iron in transcription

Multiple transcription factors and co-regulators, many of which are involved in circadian rhythm maintenance, are haem-binding proteins, although the effects of haem binding on activity appear to be inconsistent. Haem binding to the transcription factors BACH1 or CLOCK inhibits their DNA-binding capacity *in vitro* [79,80] (Figure 1), while haem promotes the activity of the transcriptional repressors, REV-ERBα and REV-ERBβ [81]. Haem binding in NPAS2 enables carbon monoxide (CO) sensing, where CO binding in the presence of haem prevents NPAS2–DNA interactions [82]. The conspicuous enrichment of circadian regulatory proteins (CLOCK, NPAS2, REV-ERBα, and REV-ERBβ) among the limited number of haem-binding transcription-associated proteins suggests that haem itself or haem-binding ligands, such as O_2_, CO, or nitric oxide (NO), are critical regulators of the circadian system. Plasma iron concentrations display circadian variation [83], implying that cellular iron also likely varies with periodicity. Furthermore, mouse models suggest that cellular iron availability influences circadian regulation [84]. Whether circadian rhythms are influenced in humans under physiological iron deficiency is less clear. However, brain iron deficiency has been linked to the circadian disorder, restless leg syndrome, which is a compulsion to move one’s legs when at rest, typically exacerbated at night [85]. Such links could plausibly relate to the effects of iron on transcriptional control of circadian regulation and/or on neurotransmitter activity (described in the ‘Iron in the nervous system’ section).

### Iron in mRNA processing and translation

Transcribed mRNAs are trafficked into the cytosol for processing and translation. Translational efficiency and fidelity are controlled at several steps by iron-dependent enzymes. Post transcription, pre-mRNAs are spliced and accumulate exon junction complexes (EJCs) at exon–exon boundaries [86]. As the resulting mRNAs are translated, EJCs are displaced by the ribosome and, once translation terminates, the Fe-S cluster protein, ABCE1, is recruited to release the ribosome from the mRNA [86] (Figure 1). In mRNAs with premature stop codons, EJCs are inefficiently removed, and nonsense-mediated decay (NMD) is initiated, preventing accumulation of deleterious truncated proteins [86]. In cells lacking ABCE1, or with impaired iron uptake, ribosomes are inefficiently recycled, resulting in aberrant ribosomal readthrough, EJC displacement, and accumulation of nonsense transcripts [86], indicating that iron is critical for transcriptome surveillance.

mRNA and tRNA demethylation are mediated by iron-dependent 2-OGDD demethylases (mRNA demethylases include FTO and ALKBH5; tRNA demethylases include TYW5, ALKBH7, and ALKBH8) and have important roles in mRNA folding, maturation, nuclear export, translation, and decay [73,87] (Figure 1). For instance, ALKBH7 demethylates mitochondrial polycistronic RNAs within the pre-Ile and Leu tRNA regions, promoting correct tRNA processing and, consequently, normal mitochondrial translation and function [88]. Finally, ribosomal hydroxylation by the 2-OGDD protein OGFOD1 is important for translation efficiency since OGFOD1 knockout results in translational stress [89]. The iron sensitivity of 2-OGDD enzymes involved in histone demethylation suggests that 2-OGDD enzymes modulating RNA methylation may be similarly iron sensitive.

Translational efficiency also depends on the addition of a hypusine moiety to the translation initiation factor, eIF5A [90]. The iron-dependent enzyme **deoxyhypusine monooxygenase (DOHH)** mediates hypusination, whereas iron chelation impairs it [91] (Figure 1). Defects in this process due to mutations in the other essential hypusination protein, DHPS, result in neuromuscular defects [90]. Whether eIF5a dysfunction during iron deficiency also contributes to neurological symptoms remains unexplored.

In summary, iron is involved in multiple steps across gene regulation, from DNA synthesis, replication, and repair, and epigenetic control, to certain transcription factors related to circadian maintenance, mRNA processing and translation. Of these processes, current evidence suggests that DNA synthesis and histone demethylation activities are most iron sensitive, although these effects have yet to be demonstrated conclusively *in vivo*. Further work is required to explore other potential effects of iron starvation, occurrence *in vivo* under physiological iron deficiency, and effects on health, particularly in the context of cancer, where gene regulation is disrupted.

## Iron in mitochondria

Iron-interacting proteins are particularly enriched in mitochondria, where 7% of mitochondrial proteins are predicted to bind iron relative to 2% overall for all proteins [6]. To cross the outer mitochondrial membrane (OMM), iron may pass through large porins called VDACs, which enable uptake of metabolites, including pyruvate and ATP [92]. Alternatively, iron may enter via direct fusion of iron-containing endosomes with the OMM [93] or via an OMM-localised isoform of DMT1 [94,95]. Once iron has entered the intermembrane space, iron is transported into the matrix via mitoferrin 1 and 2 (MFN1/2) [96,97].

**Figure 2:**
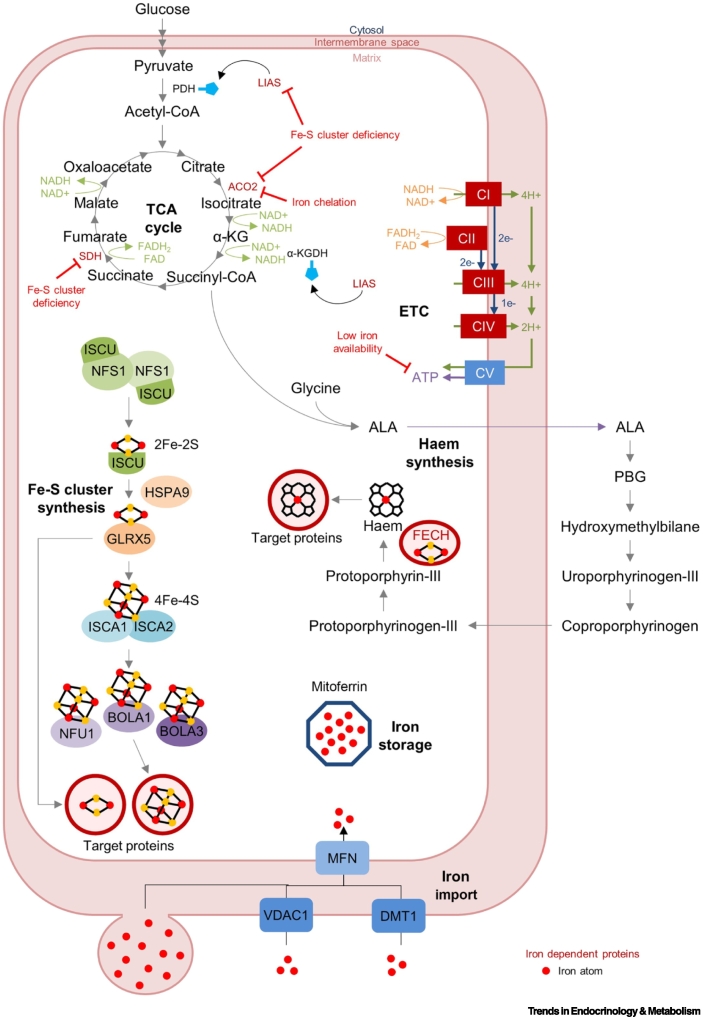
Iron in mitochondrial function. Iron crosses the outer mitochondrial membrane (OMM) into the intermembrane space by direct fusion of endocytic vesicles, via VDAC1 channels or using the DMT1 transporter. Iron then enters the matrix via mitoferrins (MFN). Iron in mitochondria is used for catalysis by aconitase 2 (ACO2) and succinate dehydrogenase (SDH) in the tricarboxylic acid (TCA) cycle, and by lipoic acid synthase (LIAS) to deposit lipoic acid on the enzymes pyruvate dehydrogenase (PDH) and α-KG dehydrogenase (α-KGDH). Iron cofactors are required for electron transfer by all four complexes of the electron transport chain (ETC), and for haem and iron sulfur (Fe-S) cluster synthesis. Succinyl-CoA from the TCA cycle can be condensed with glycine to initiate haem synthesis, which is an eight-step process that concludes with the deposition of iron into the porphyrin ring structure by the [2Fe-2S] cluster-binding protein ferrochelatase (FECH). Fe-S cluster synthesis begins with the formation of a [2Fe-2S] cluster on a scaffold of proteins, which includes ISCU and NFS1. The [2Fe-2S] cluster is transferred to glutaredoxin 5 (GLRX5) with the help of HSPA9 [108]. GLRX5 can transfer the [2Fe-2S] cluster to target proteins or two can be linked together to form a [4Fe-4S] cluster on the Fe-S cluster assembly 1 and 2 (ISCA1/)2 complex. [4Fe-4S] clusters are delivered to target proteins by chaperones, such as BOLA1, BOLA3 and NFU1. Iron-dependent proteins are denoted in dark red. Green text shows production of NADH/FADH_2_, while orange text shows consumption of NADH/FADH_2_. Red dots denote iron atoms.

Mitochondrially localised iron can be stored in mitochondrial ferritin [98,99], utilised for haem and Fe-S cluster biosynthesis, or used by mitochondrial proteins [100] (Figure 2). Inefficient haem or Fe-S cluster synthesis due to mutations in these pathways causes mitochondrial iron overload as cells attempt to rectify biosynthetic blocks by trafficking further iron into the mitochondria [100,101]. Notably, this mitochondrial iron loading comes at the expense of cytosolic iron homeostasis, resulting in cytosolic iron deficiency and induction of the low iron IRP–IRE response, suggesting that, as destinations for iron, mitochondria are hierarchically above other cellular compartments [101].

Mitochondria are particularly sensitive to oxidative stress due to the presence of the electron transport chain (ETC) machinery and other iron-dependent redox processes. Iron-related lipid oxidation is particularly implicated in ferroptosis, a nonapoptotic cell death pathway [15,102] (Box 1). Glutathione (GSH) is a cytosolically synthesised antioxidant imported into the mitochondria by the Fe-S cluster-binding protein, SLC25A39, to aid mitochondrial antioxidant responses [103,104]. SLC25A39 import is regulated by iron: during iron scarcity and in the absence of [2Fe-2S] cluster binding, SLC25A39 is degraded by AFG3L2 [103,104]. During iron repletion, SLC25A39 binds a [2Fe-2S] cluster, promoting its stabilisation and GSH mitochondrial import [103,104], demonstrating tight links between iron and the antioxidant response.

Box 1 **Iron in cellular toxicity and ferroptosis**

The capacity of iron to easily exchange electrons has been capitalised on by many cellular proteins and enzymes. However, these same redox properties that make iron useful also contribute to cellular toxicity by driving Fenton reactions and the production of free radicals, which can damage cellular components [13]. Iron overload, for instance in hereditary hemochromatosis, can result in a multi-tissue disease with toxicity in organs, including the liver (liver cirrhosis and hepatocellular carcinoma), heart, and pancreas (diabetes) [223].

Beyond simply causing cellular damage via Fenton reactions, iron has also more recently been implicated in an alternative, regulated, nonapoptotic cell death pathway known as ferroptosis [102]. Ferroptosis is characterised by the peroxidation of PUFA-containing phospholipids [102]. These phospholipids are specifically implicated because the double bonds contained within the PUFA chains make these phospholipids particularly susceptible to oxidation damage [102]. Mutations in genes preventing PUFA usage in phospholipids are protective against ferroptosis [224., 225., 226., 227.]. While the term ‘ferroptosis’ implies the requirement of iron for this process, it is unclear how iron specifically mediates lipid peroxidation [15]. Previous work attributed lipid peroxidation activity to iron itself, iron-containing lipoxygenase enzymes, and haem-dependent CYB5R1 [15]. Under homeostatic conditions, lipid peroxidation is controlled by lipid peroxide repair mechanisms [102]. GPX4 appears to be the main enzyme involved in lipid oxidation detoxification and utilises glutathione to convert lipid peroxides to lipid alcohols [102]. Vitamin E and coenzyme Q10 have also been shown to detoxify lipid peroxides [102]. Accumulation of sufficient lipid peroxides results in membrane rupture and death [228].

Alt-text: Box 1

### Iron in haem and Fe-S cluster biosynthesis

Haem synthesis begins with condensation of glycine to succinyl-CoA by the rate-limiting enzymes, ALAS1/2 (ALAS1 is found in all cells, while ALAS2 is erythroid lineage specific) in the mitochondrial matrix [100,105] (Figure 2). The product, 5-aminolevulinic acid (ALA), passes through seven cytosolic (steps 2–5) and mitochondrial (steps 6–8) reactions. Insertion of iron into porphyrin by the Fe-S cluster-containing protein, ferrochelatase (FECH) completes haem production [100,106]. Haem can be used locally [e.g., in complex I (CI) of the ETC] or transported to the cytosol by FLVCR1b for other uses in the cell [100,107].

Fe-S cluster synthesis includes both mitochondrial and cytosolic assembly pathways [108]. Mitochondrial synthesis begins with formation of a scaffold including two each of the proteins ISCU2, NFS1, frataxin, FDX2, ISD11, and ACP1 [108] (Figure 2). This complex extracts sulfur from the amino acid cysteine and combines it with iron, forming a [2Fe-2S] cluster [108]. ISCU2 transfers [2Fe-2S] to a GLRX5 dimer, assisted by HSPA9 (HSP70) and HSP40 (HSC20) [108]. [2Fe-2S] clusters can either be delivered to proteins by GLRX5 or fused to form [4Fe-4S] clusters by a complex comprising **Fe-S cluster assembly 1 and 2 (ISCA1/)2** and IBA57 [108]. [4Fe-4S] clusters are then inserted into their target proteins by chaperones, including NFU1, IND1, BOLA1, and BOLA3 [108]. Mitochondrial Fe-S cluster targets include ACO2, LIAS, and CI and complex II (CII) of the ETC [108].

Although intact mitochondrial Fe-S cluster synthesis is essential for nonmitochondrial Fe-S cluster-binding proteins, mitochondrially derived Fe-S clusters are not believed to cross the mitochondrial membrane [108]. Instead, an uncharacterised sulfur-containing factor (X-S) is thought to be produced by mitochondrial Fe-S cluster synthesis and passaged to the cytosol by ABCB7 for completion by the cytosolic Fe-S cluster synthesis pathway [108].

The essentiality of Fe-S clusters for cellular function is demonstrated by individuals with Fe-S cluster biogenesis defects caused by mutations in genes encoding frataxin, GLRX5, and ISCU [109]. Mutations in frataxin result in Friedrich’s ataxia, a neurodegenerative disease featuring limb ataxia, muscle weakness, and cardiomyopathy [109]. Meanwhile, GLRX5 and ISCU deficiencies cause microcytic anaemia and myopathy, respectively [109]. Interestingly, each of these mutations presents with different predominant clinical effects despite similar underlying biochemical features, including dysfunction in enzymes, such as ACO2, and cellular iron loading [109]. Iron deficiency could conceivably modulate phenotypic severities of mutations in Fe-S cluster biosynthesis.

### Iron in mitochondrial metabolism

Mitochondrial metabolism is highly iron dependent. ACO2 and succinate dehydrogenase (SDH) use Fe-S clusters to catalyse essential steps in the TCA cycle, which generates metabolic intermediates and the redox cofactors, NADH and FADH_2_ [100] (Figure 2). Electrons from NADH and FADH_2_ are passed to four ETC complexes that heavily rely on haem and Fe-S clusters for efficient electron transfer [100]. The energy released from the ETC generates a proton gradient across the inner mitochondrial membrane (IMM) that promotes ATP production [100]. Beyond energy generation, a [4Fe-4S] cluster in LIAS mediates the essential lipoylation of metabolic enzymes, such as pyruvate dehydrogenase (PDH) and α-KG dehydrogenase (α-KGDH) [108]. **Electron transfer flavoprotein dehydrogenase (ETFDH)** and FECH use Fe-S clusters for fatty acid β-oxidation and the final step of haem synthesis, respectively [108], and METTL17 utilises a [4Fe-4S] cluster in mitochondrial ribosome assembly and translation [110].

The high dependence of mitochondria on iron for metabolic processes and haem and Fe-S cluster synthesis suggests that iron deficiency profoundly affects mitochondrial processes and extra-mitochondrial pathways that rely on haem, Fe-S cluster cofactors, or mitochondrially derived metabolites produced by iron-dependent processes (e.g., α-KG for 2-ODGG enzymes).

Accordingly, swollen, rounded mitochondria with aberrant cristae and fewer mitochondrial cytochromes are observed under iron-deficient conditions [111., 112., 113.]. Liver mitochondria from iron-deficient rats have decreased mitochondrial respiration efficiency [114], lower mitochondrial membrane potential and ATP production [113], and suppressed capacity for gluconeogenesis via **phosphoenolpyruvate carboxykinase (PCK)** [115]. Similarly, T cell iron restriction abrogates mitochondrial ATP production and reduces mitochondrial membrane potential [59,116,117]. Iron chelation also impairs TCA cycle progression, with citrate and succinate accumulation upstream of iron-dependent ACO2 and SDH and depletion of the downstream metabolites, fumarate and malate [51]. As noted in the preceding text, iron deficiency can induce shifts toward glycolysis; none of the enzymes in the core glycolysis pathway are iron dependent. ISCU2 mutant cells lacking efficient Fe-S cluster synthesis similarly accumulate citrate due to reduced ACO2 activity, accompanied by impaired SDH and LIAS activity, and elevated fatty acid synthesis and lipid droplet formation [118].

Together, it appears likely that altered mitochondrial function and metabolism resulting from iron deficiency influences cells and tissues in humans, and contributes to disease processes; however, this is not well characterised.

## Iron in lipid metabolism

Metabolism of lipids, including fatty acids, phospholipids, cholesterol and its derivatives (steroid hormones, bile acids, and vitamin D), and eicosanoids [119], involves many iron-dependent oxidoreductase enzymes, which utilise iron to enable complex metabolic reactions.

### Iron in cholesterol metabolism

**Figure 3:**
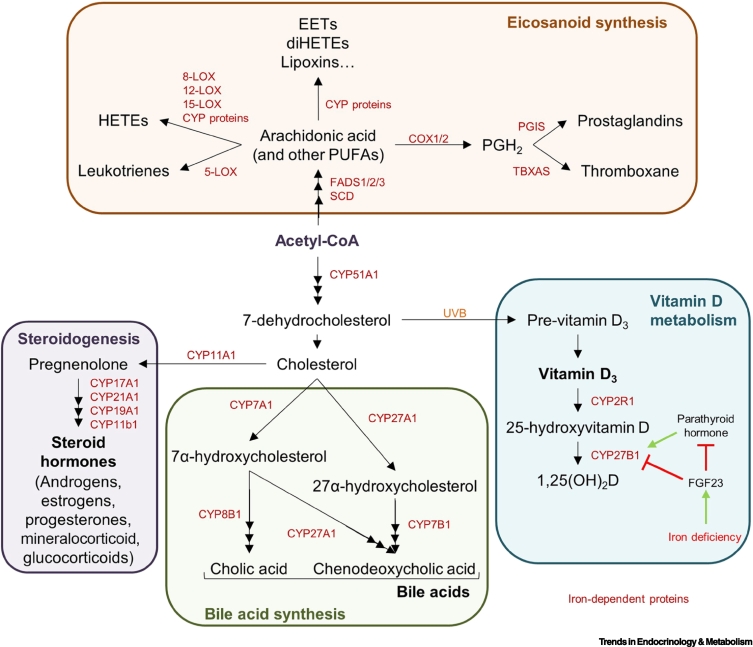
Iron in lipid metabolism. Iron is utilised for the synthesis and metabolism of a diversity of lipid species. Polyunsaturated fatty acids (PUFAs), such as arachidonic acid, are synthesised from acetyl-CoA using the iron-dependent enzymes fatty acid desaturase (FADS)-1/2/3 and stearoyl-CoA desaturase (SCD), to mediate the desaturation reactions. Arachidonic acid is used for eicosanoid metabolism. Cyclooxygenase 1/2 (COX1/2) uses iron to convert arachidonic acid to PGH_2_ which is subsequently metabolised to prostaglandins and thromboxane by cytochrome P450 (CYP) proteins. The lipoxygenase (LOX) enzymes [(5-LOX, 8-LOX, 12-LOX, and 15-LOX) use iron to produce leukotrienes and hydroxyeicosatetraenoic acids (HETEs). The CYP proteins use haem cofactors to mediate synthesis of HETES, epoxyeicosatrienoic acids (EETs), dihydroxyeicosatetraenoic acid (diHETEs), and lipoxins. Acetyl-CoA is also converted to cholesterol via a pathway requiring haem-dependent CYP51A1. CYP proteins convert cholesterol to steroid hormones in tissues, such as the adrenal glands, testes, and ovaries, and to bile acids in the liver. The cholesterol precursor 7-hydrocholesterol is also converted to vitamin D via sunlight and is processed to the active form, 1,25(OH_2_)D by haem-dependent CYP2R1 and CYP27B1. Iron deficiency induces production of the growth factor FGF23, which inhibits the activity of CYP27B1 directly and via suppression of the CYP27B1 agonist, parathyroid hormone. Iron-dependent proteins are denoted in dark red.

Cholesterol is generated endogenously or can be acquired from exogenous sources via uptake of low-density lipoproteins (LDLs) containing cholesteryl ester [120]. Endogenous cholesterol synthesis is a complex multistep process catalysed by many enzymes, including the haem-dependent enzyme, CYP51A1, which metabolises the early cholesterol precursor, lanosterol [121] (Figure 3). Downstream cholesterol metabolism, which is critical for the synthesis of steroid hormones, bile acids, and vitamin D, depends on haem-dependent cytochrome P450 enzymes [**cytochrome P450 (CYP) proteins**] [122].

CYP proteins use haem prosthetic groups in metabolising diverse targets, including endogenous fatty acids, bile acids, and steroids, and for detoxifying xenobiotics, such as pharmaceuticals [122]. CYP proteins are classed as mitochondrial-localised type 1 and endoplasmic reticulum (ER)-localised type 2 CYP proteins [123]. Type 1 CYP proteins rely on electrons passed from NADPH via ferrochelatase to the Fe-S cluster protein ferredoxin [123], which then passes electrons to the CYP proteins [123]. The ferredoxin dependence of type 1 CYP proteins renders mitochondrial CYP reactions dually iron dependent, relying on both haem and Fe-S cluster cofactors. Type 2 CYP proteins contrastingly receive electrons from NADPH via the protein POR [123].

The sex-linked anaemic (SLA) mutation in mice prevents intestinal iron absorption by impairing the ferroxidase hephaestin, causing iron deficiency anaemia [124]. SLA mice have reduced hepatic cholesterol synthesis [124]. Iron chelators suppress cholesterol levels in neuroblastoma cell lines [125], while, conversely, elevated iron availability promotes cholesterol synthesis in endothelial cells [126]. Together, this suggests that iron availability modulates cholesterol metabolism. Whether this relates to direct impairment of cholesterol synthesis via iron-dependent CYP51A1 or to negative feedback on cholesterol biosynthesis by downstream iron-dependent pathways is unclear. Although mechanistic links between iron regulation and cholesterol levels are evident in mice [127], further work should explore how altered iron influences cholesterol metabolism in humans.

### Iron in steroid hormone synthesis

Steroid hormones are synthesised from cholesterol and have many roles in systemic biology. Five classes of steroid hormone exist: androgens (e.g., testosterone), oestrogens (e.g., oestradiol), progestogens (e.g., progesterone), glucocorticoids (e.g., cortisol), and mineralocorticoids (e.g., aldosterone) [128]. Androgens, oestrogens, and progestogens are involved in reproductive biology, with roles such as sex differentiation and pregnancy maintenance [123,128]. Glucocorticoids and mineralocorticoids regulate cell metabolism and salt and water balance, respectively [128].

Steroid hormone synthesis begins with cholesterol transport into the mitochondria for conversion to pregnenolone by haem-dependent CYP11A1 [123] (Figure 3). The various steroid hormones are then synthesised by haem-dependent CYP proteins, which mediate irreversible hydroxylation reactions and hydroxysteroid dehydrogenases (HSD), which catalyse reversible reactions [123]. Although cortisol secretion may be reduced in iron deficiency in humans [129], establishing whether physiological iron deficiency impacts steroid synthesis, with potential implications for development and reproductive fitness, requires more work.

### Iron in vitamin D metabolism

Vitamin D promotes calcium and phosphorous absorption for skeletal development [130]. It comes in two forms, with different sidechains but similar behaviour [131]. Vitamin D_2_ derives from plant and fungal dietary sources, while vitamin D_3_ is synthesised in the skin from the cholesterol precursor, 7-dehydrocholesterol (7-HDC), which is non-enzymatically converted to pre-vitamin D_3_ by ultraviolet (UV)-B radiation [131]. Pre-vitamin D_3_ subsequently isomerises to vitamin D_3_ [131] (Figure 3). For biological activity, vitamin D must be converted to 25-hydroxyvitamin D (25-OHD) and then to 1,25(OH)_2_D [131] in reactions mediated by the haem-dependent enzymes CYP2R1 (in the liver) and CYP27B1 (in the kidney, but also in keratinocytes and immune cells), respectively [131]. Similar to Vitamin D deficiency, mutation of either enzyme results in rickets, where defective bone mineralisation causes skeletal deformities, indicating the crucial role of these iron-dependent enzymes in vitamin D metabolism [130,131].

CYP27B1 activity is inhibited by FGF23 [131]. Notably, FGF23 is upregulated during iron deficiency, resulting in CYP27B1 downregulation and suppressed 1,25(OH)_2_D production [132] (Figure 3). FGF23 also indirectly suppresses 1,25(OH)_2_D production via suppression of parathyroid hormone, which stimulates CYP27B1 [132]; however, whether iron scarcity directly impairs CYP27B1 activity is unclear. Notably, reduced iron intake has been associated with reduced bone mineral density [133] and increased risk for osteoporosis [134] in women. Iron deficiency in rats also significantly reduces bone mineral content and density [135,136]. These data suggest that iron deficiency may impair bone formation potentially via suppression of vitamin D synthesis, but this requires further investigation.

### Iron in bile acid metabolism

Bile acids are synthesised exclusively in the liver from cholesterol and are excreted in bile with cholesterol and phospholipids into the small intestine following food ingestion [137]. Bile acids act as detergents facilitating solubilisation, digestion and absorption of lipophilic molecules, including dietary fats, steroids, certain drugs, and vitamins (A, D, E, and K) [137]. In humans, most bile acids are synthesised via the classical pathway (>90%), which is initiated by ER-localised haem-dependent CYP7A1, but also requires CYP8B1 and CYP27A1 [137] (Figure 3). An alternative pathway utilising CYP27A1 and CYP7B1 also generates bile acids (<10%) [137]. Reduced bile acid synthesis during iron overload may partly result from CYP7A1 downregulation [138], but whether direct effects of iron on bile acid metabolism or general liver dysfunction are responsible is unclear. Interestingly, a study in prairie dogs found that iron-deficient animals had lower CYP7A1 levels and increased likelihood of cholesterol gallstones [139]. Cholesterol gallstones form when cholesterol becomes insoluble in bile; some bile acids promote cholesterol solubility and protect against gallstones, consistent with impaired bile acid synthesis promoting gallstones [140]. Notably, an increased frequency of gallstones in patients with iron deficiency has also been reported [141]; however, these potential mechanistic links require confirmation.

### Iron in fatty acid and eicosanoid metabolism

Most enzymes involved in fatty acid synthesis do not utilise iron. However, the fatty acid desaturase enzymes, **fatty acid desaturase (FADS)-**1/2/3 and **stearoyl-CoA desaturase (SCD)**, which generate **polyunsaturated fatty acids (PUFAs**; i.e., double bond-containing), rely on di-iron cores [119] (Figure 3). PUFAs are used in several downstream metabolic processes, including sphingolipid and eicosanoid synthesis [119,142]. PUFAs and their derivatives influence membrane fluidity and cellular signalling, as both lipid mediators and agonists for nuclear receptor transcription factors, including PPAR and LXR [142]. Notably, rats fed an iron-deficient diet displayed decreased plasma levels of the PUFA arachidonic acid [143] and reduced hepatic PUFAs in phosphatidylcholine and phosphatidylethanolamine phospholipids, suggesting reduced desaturase activity [144,145]. Whether iron deficiency similarly suppresses fatty acid desaturation in humans with impacts on downstream processes, such as membrane remodelling and signalling, remains unexplored.

Arachidonic acid metabolism is critical for synthesis of eicosanoid lipid mediators, such as prostaglandins, thromboxane, and leukotrienes [146]. Eicosanoids have important roles in cardiovascular function, inflammation, and cancer and are metabolised from arachidonic acid by three classes of iron-dependent enzyme [146] (Figure 3). The iron-dependent lipoxygenase (LOX) enzymes catalyse leukotriene and hydroxyeicosatetraenoic acid (HETE) synthesis [146]. Meanwhile, the cyclooxygenases (COX1/2), which are targets of nonsteroidal anti-inflammatory drugs (NSAIDs), such as ibuprofen and aspirin, are haem dependent, catalysing the initial step in prostaglandin and thromboxane synthesis [146,147]. COX enzymes convert arachidonic acid to PGH_2_ and PHG_2_, which are metabolised to a variety of prostaglandins, including by the iron-dependent CYP proteins PGIS (CYP8A1) and TBXAS (CYP5A1), yielding prostacyclin (PGI_2_) and thromboxane A_2_ (TXA_2_), respectively [146]. CYP proteins are also involved in the production of HETEs, epoxyeicosatrienoic acids (EETs), dihydroxyeicosatetraenoic acid (diHETEs), and lipoxins [146].

Given the important role of eicosanoids in settings such as inflammation and cardiovascular function, alterations in eicosanoid metabolism could have important implications for human health. Iron-overloaded *in vitro*-cultured myocytes produce higher levels of the prostaglandins PGE_2_, PGF_2α_, and PGI_2_ [148]_,_ suggesting that eicosanoid synthesis is iron sensitive.

## Iron in cellular detoxification and catabolism

Iron-dependent proteins have key roles in drug metabolism, detoxification, and catabolism of cellular components. Many haem-dependent CYP proteins effect drug detoxification [122], and THAP4 and catalase use haem to detoxify the reactive nitrogen and oxygen species, peroxynitrite and H_2_O_2_, respectively [149,150]. By contrast, **phytanoyl-CoA dioxygenase (PHYH)** is a 2-OGDD enzyme that degrades dietary phytanic acid [151]. Degradation of specific amino acids similarly requires iron. Tyrosine degradation requires iron-dependent **4-hydroxyphenylpyruvate dioxygenase (HPD)** and **homogentisate 1,2-dioxygenase (HGD)** [152]. Meanwhile, three iron-dependent enzymes, **tryptophan 1,2-dioxygenase (TDO)**, **indoleamine 2,3-dioxygenase 1/2 (IDO1/2)**, initiate tryptophan degradation, converting tryptophan to *N*-formyl-kynurenine [153]. Iron-dependent **3-hydroxyanthranilate 3,4-dioxygenase (HAAO)** acts downstream in tryptophan degradation [153]. Decreased serum tryptophan has been described during iron deficiency [154], but whether this relates directly to tryptophan metabolism and/or degradation is unclear.

Three critical degradative enzymes, SUOX, **xanthine dehydrogenase (XDH),** and AOX1, require not only iron, but also molybdenum, another essential micronutrient [155]. Molybdenum is always complexed within the molybdenum cofactor (MOCO), a complex nitrogen heterocyclic structure that requires iron for its biosynthesis [155]. Specifically, MOCS1A uses two [4Fe-4S] clusters to initiate MOCO synthesis [155]. SUOX uses MOCO and haem to convert toxic sulfite released from cysteine degradation to sulfate [156]. While SUOX deficiency typically results in toxic accumulation of sulfite causing death in infancy, a novel SUOX mutation in the haem-binding domain (SUOX^H143N^) associated with milder disease was recently identified [157]. Despite loss of the haem cofactor, SUOX^H143N^, still retains capacity to detoxify sulfite [157], suggesting that iron deficiency would only have mild effects on SUOX function. XDH uses MOCO and two [2Fe-2S] clusters to convert hypoxanthine produced from purine degradation to uric acid [156]. Interestingly, dietary iron deficiency suppresses intestinal, but not liver XDH activity in rats [158]. Similar to XDH, AOX1 requires MOCO and two [2Fe-2S] clusters, but mediates the conversion of aldehydes to carboxylic acids on a range of substrates, making it important for drug metabolism [156].

The extensive use of iron for detoxification and catabolic reactions suggests that iron starvation results in accumulation of toxic compounds. Iron-deficient rats may display defective hepatic detoxification [159], but the extent and specificity of the impact of iron deficiency on catabolism remain uncertain.

## Iron in tissue and cell-specific processes

### Iron in the immune system

Iron is increasingly recognised to have important roles in immune function. Most prominently, children bearing mutations in the gene encoding the iron uptake receptor, TFRC, have impaired adaptive and innate immune responses, presenting as combined immunodeficiency; these mutations cause impaired cellular iron uptake [160., 161., 162.]. Low serum iron is associated with nonresponse to influenza virus vaccination in older individuals [163], while iron supplementation in Kenyan infants who were likely iron deficient increased vaccine responses [12], pointing to interactions between iron deficiency and vaccine efficacy [164].

**Figure 4:**
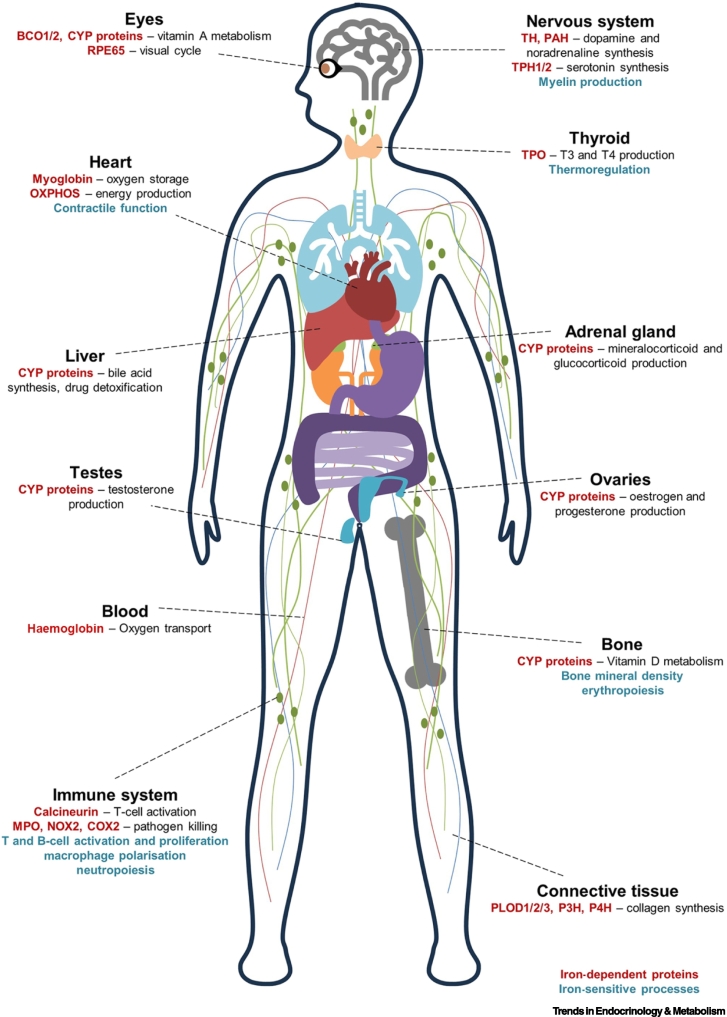
Tissue and system specific roles of iron. Iron is utilised by all mammalian cells. However, iron-interacting proteins have many cell-, organ-, and tissue-specific roles. Bold red text denotes known iron-dependent proteins. Bold blue text denotes described iron-dependent processes with incomplete mechanistic understanding. Abbreviations: COX2, cyclooxygenase; CYP, cytochrome P450; MPO, myeloperoxidase; OXPHOS: oxidative phosphorylation; PAH, phenylalanine hydroxylase; PLOD, procollagen lysine 2-oxoglutarate 5-dioxygenase; T3, triiodothyronine; T4, thyroxine; TH, tyrosine hydroxylase; TPH, tryptophan hydroxylase; TPO, thyroid peroxidase.

Innate immune cells, including macrophages, neutrophils, and natural killer (NK) cells rapidly respond to infections or perturbations [165]. Macrophages and neutrophils facilitate bacterial clearance by phagocytosis and utilise multiple iron-requiring enzymes, including **Myeloperoxidase (MPO)**, NOX2, and COX2, for pathogen killing via production of toxic ROS and reactive nitrogen species [166., 167., 168., 169.] (Figure 4). Iron-depleted neutrophils show defects in phagocytosis, bacterial killing, and cytokine production [170], but the specific cellular pathways that are impaired are unclear. Moreover, neutrophil development is also impaired by iron restriction [170,171]. Meanwhile, differential expression of key iron regulatory proteins associates with macrophage polarisation toward inflammatory or reparative subsets [172]. Proinflammatory M1 macrophages are more iron sequestering (reduced ferroportin, increased ferritin), while tissue homeostatic M2 macrophages are primed for iron export (increased ferroportin, reduced ferritin) [172]. Correspondingly, iron loading drives inflammatory macrophages, while iron chelation attenuates inflammatory signatures [173,174]. NK cells, which are early-responding cytotoxic cells important for killing virus-infected cells and tumour cells [175], are also impaired by iron starvation [176].

The adaptive immune arm comprising B and T cells provides higher specificity responses to immune stimuli together with immune memory [177]. T cell receptor (TCR) binding to specific cognate antigen:MHC complexes induces activation via signalling pathways involving calcium influx [178]. Calcium activates calmodulin, which signals via iron-dependent calcineurin to stimulate the transcription factor NFAT [178,179] (Figure 4). Whether iron scarcity impairs calcineurin signalling is unclear. However, iron depletion does impair T and B cell responses to vaccination [59,77], influenza infection [59], and autoimmune stimuli [117,180., 181., 182.]. Iron-starved T cells fail to proliferate, show defects in mitochondrial function [59], and have increased levels of the repressive histone mark H3K27me3 [78]. Iron-starved B cells similarly show proliferative defects and suppressed removal of H3K9me2/3 [77]. Overall, while it is clear that iron scarcity impacts immunity at both the cellular and systems levels, whether this is driven by one or more specific requirements of cellular function for iron (e.g., DNA synthesis, mitochondrial function, or epigenetic regulation) remains to be determined. At a higher level, how iron deficiency quantitatively and qualitatively influences immune system development (e.g., haematopoietic output, and immune cell populations at barrier sites) requires more research.

### Iron in the nervous system

Iron deficiency is commonly associated with fatigue, impaired cognitive development, and dizziness [10]. While anaemia and reduced oxygen availability may contribute to this, iron may also directly influence neurological function [183]. Serotonin production from tryptophan requires the iron-dependent enzyme **tryptophan hydroxylase 1/2 (TPH1/2)** [184] (Figure 4). Meanwhile, dopamine and noradrenaline synthesis from phenylalanine (via tyrosine) requires iron-dependent **tyrosine hydroxylase (TH)** and **phenylalanine hydroxylase (PAH)** [184]. Serotonin, dopamine, and noradrenaline are neurotransmitters that regulate neurological processes, including emotional state [184]. Notably, restless leg syndrome, believed to be caused by dysregulated dopamine signalling, is associated with brain iron deficiency [10,185].

Early-life iron deficiency in rats was shown to alter DNA and histone methylation at the gene encoding **brain-derived neurotrophic factor (BDNF)**, important for neural function in the hippocampus [186]. Iron is also implicated in myelin-producing oligodendrocytes, which have high requirements for OXPHOS [187]. Such factors may underly well-established associations between iron deficiency and impaired cognitive development in infants [188].

### Iron in thyroid function

**Thyroid peroxidase (TPO)** is an iron-dependent enzyme important for production of the thyroid hormones triiodothyronine (T3) and thyroxine (T4) [189] (Figure 4). Thyroid hormones control systemic metabolism, with important roles in thermoregulation [190]. Upon cold exposure, they stimulate heat preserving and generating pathways, such as vasoconstriction, shivering, and activation of heat-generating brown adipose tissue [190]. Rats on iron-deficient diets have reduced TPO activity and thyroid hormone production [191] and fail to upregulate thyroid hormones upon cold exposure [192]. Lower T3 and T4 hormone levels and significantly lower body temperatures upon cold exposure are also reported in women who are iron deficient, suggesting impaired thermoregulation [193,194].

### Iron in collagen synthesis

Collagens comprise ∼30% of the protein mass of the body, maintaining integrity of tissues, such as skin and tendons [195]. They require numerous post-translational modifications [195]. For instance, iron-dependent 2-OGDD enzymes [**procollagen lysine 2-oxoglutarate 5-dioxygenase (PLOD)**1/2/3, P3H, and P4H] mediate proline and lysine hydroxylation [195,196] (Figure 4). Collagens are also cleaved and experience cross-linking between different collagen strands [195]. Type 1 collagen is a major component of bone [133,135,136]; thus, changes in bone density with altered iron (discussed in the preceding text) may also involve defects in collagen synthesis. Koilonychia (indented nails) and dry skin are also associated with iron deficiency [10], but whether these relate to impaired collagen synthesis is unclear.

### Iron in vitamin A metabolism and vision

Vitamin A (retinol) cannot be synthesised endogenously by mammals and is instead acquired from the diet as retinyl esters or β-carotene [197]. Iron-dependent BCO1 mediates the initial cleavage of dietary β-carotene to retinal, before conversion to retinol, retinyl esters, and retinoic acid (Figure 4) [197]. Retinal is important for vision, while retinoic acid stimulates transcription by retinoic acid receptor transcription factors, which control diverse processes, including immunity, cell differentiation, and development [197]. Retinoic acid clearance depends on haem-dependent CYP26 proteins [198], implicating iron in both vitamin A synthesis and degradation. Iron deficiency in rats associates with reduced plasma retinol and increased liver retinol, consistent with iron influencing vitamin A mobilisation [199,200]. Note that plasma vitamin A, similar to iron, is negatively regulated upon inflammation due to increased liver retention [201], driving correlations between iron and vitamin A, especially during inflammation.

Upon light exposure, photon absorption in the retina converts 11-*cis*-retinal to all-*trans*-retinal, inducing a phototransduction cascade and, consequently, vision [202]. For continuous vision, all-*trans*-retinal must be converted back to 11-*cis*-retinal in the retinal pigment epithelium (i.e., the visual cycle) [202], requiring the iron-dependent enzyme RPE65 [202]. Mutations in the histidines that allow iron binding, or iron chelation, abrogate RPE65 function [202,203]. Interestingly, the iron chelator deferoxamine has been associated with ocular toxicity [204].

### Iron in muscle and cardiovascular function

Iron deficiency is highly prevalent in patients with heart failure, and serum iron deficiency associates with increased risk of cardiovascular disease and worse outcomes upon heart failure [121] (Figure 4). In mouse models, cardiomyocyte iron deficiency has been linked with cardiomyopathy and impaired contractile function [205,206], while maternal iron deficiency has been implicated in severe embryonic cardiovascular defects [207]. The mechanisms driving these clear iron–cardiovascular interactions are uncertain. Similar to haemoglobin, myoglobin uses iron to store oxygen in cardiac and skeletal muscle [121] and iron-deficient rats show reduced myoglobin in skeletal muscle, but not cardiac muscle [208]. Muscle also has high metabolic demands and, accordingly, iron deficiency in cardiomyocytes promotes dysfunctional mitochondrial processes, including impaired ETC activity, increased oxidative stress, and a switch to glycolysis [205,206,209,210]. Iron deficiency in rats similarly results in muscle cytochrome C depletion and reduced activity of CI and CII (SDH) [211].

## Concluding remarks and future perspectives

We have described many roles of iron in mammalian cellular biochemistry and summarised what is known regarding the impacts of iron perturbations on these processes. Clearly, our review cannot be exhaustive; other iron-dependent proteins exist in humans [6] and especially in other organisms, and others may remain to be discovered. However, despite the extensive involvement of iron in cellular biochemistry, the mechanisms by which iron deficiency interacts with these processes are, in many cases, either uncharacterised or definitive data remain somewhat limited. Nevertheless, understanding where and how iron is utilised at the cellular level lays the groundwork for rationally hypothesising and testing how iron deficiency may cause dysfunction potentially affecting clinical pathology. Moreover, such understanding may suggest whether known pathological conditions have iron dysregulation implicated in their pathogenesis (see Outstanding questions).

Iron scarcity has several underlying causes in humans, ranging from dietary iron deficiency [10] and inflammation-induced hypoferremia [212] to iron restriction within local tissue niches due to the presence of iron-demanding tumours [11] or pathogens [213]. While all mammalian cells require iron, different cell and tissue types will likely have different iron requirements and, consequently, differential sensitivity to iron deficiency. Demonstrating this principle, erythroid lineages, which rely heavily on iron for haemoglobin, are more sensitive to *in vitro iron deprivation*compared with megakaryocytic and granulocytic lineages [214]. Similarly, neutropoiesis shows higher sensitivity to *in vivo* iron limitation relative to monopoiesis, potentially due to the high predicted iron requirements of neutrophils [170]. Furthermore, cells that experience high turnover will likely have higher iron demands due to cell division and dilution of iron between daughter cells. For example, activated and rapidly proliferating T cells are considerably more sensitive to iron scarcity compared with their naïve and quiescent counterparts [160]. Cells that rely heavily on iron-dependent processes, such as mitochondrial energy generation in cardiomyocytes, would also be predicted to be more affected by iron starvation.

The interaction of iron scarcity with inflammatory signals and immune cell activation and migration could influence multiple factors in cellular and tissue biology. One relevant scenario is viral infection that induces strong inflammatory responses and hypoferremia: during SARS-CoV-2 infection, plasma iron concentrations can plummet to extremely low levels because tissue damage is ongoing [215,216]. Dysregulation of iron homeostasis early in infection may impair tissue oxygenation due to decreasing haemoglobin and influence cellular metabolism via iron redistribution [217]. Indeed, altered iron homeostasis, impaired erythropoiesis, iron-deficient lymphocytes, but iron-loaded monocyte populations during the first month after SARS-CoV-2 infection are associated with symptoms of Long Covid [218]. This may be an example of disturbances of iron availability at cellular level influencing metabolism and function and leading to chronic systemic pathology [218,219].

A consideration for understanding cellular effects of iron deficiency is that iron-dependent proteins will likely have different sensitivities to iron deprivation, with distinct cellular processes likely differentially impacted by reduced iron. For instance, the proteins from which iron is sequentially lost during iron scarcity will be governed by factors including iron binding affinity and protein turnover. Notably, different 2-OGDD proteins are suggested to have different iron-binding affinities [220]. Specifically, PHD2 (involved in HIF signalling) displays very high binding affinity, whereas P4H (collagen hydroxylation) binds iron more weakly [220]. Thus, P4H may lose iron prematurely, while more extreme iron scarcity may be required to induce HIF signalling. Differential binding affinities are also likely to vary within Fe-S cluster and haem-binding protein families. Proteins experiencing slower turnover may retain their iron cofactors when iron limitation is imposed, making them more resistant to iron deprivation relative to proteins with a high rate of turnover, which will more readily recycle iron back into the cellular iron pool.

Similarly, iron trafficking to different proteins and organelles by intracellular iron chaperones will also modulate iron usage by cellular processes. It is well established that mitochondria are treated preferentially in terms of iron supply within the cell [100,101], suggesting that mitochondrial processes, such as haem and Fe-S cluster synthesis and energy generation, are favoured over other cellular processes. Moreover, certain iron-dependent proteins have redundancies with iron-independent alternatives. For instance, iron-dependent helicases represent only a fraction of the 31 DNA helicases encoded by the human genome [221], suggesting that, during iron limitation, other iron-independent helicases may intervene, reducing or completely preventing dysfunction (although note that different helicases preferentially bind certain nucleic acid structures, meaning that true redundancy between all 31 helicases is unlikely). By contrast, other proteins, such as the haem-dependent CYP proteins involved in steroidogenesis, have no known iron-independent substitutes. Understanding the different sensitivities of tissues, cell types, and proteins to iron scarcity will provide insights into how iron modulation may be used to target specific biological processes.

Iron deficiency is highly prevalent and may make underappreciated contributions to several health issues. For instance, iron deficiency is common in some forms of cancer [71] and heart failure [121], but whether and how iron deficiency contributes to poor health outcomes is not well characterised. Large-scale population studies of biobank data may provide indications of associations between iron deficiency and common health problems, which could then be interrogated mechanistically using cell and animal experimental models. Understanding the biochemical implications of iron restriction on cellular processes should inform how pharmacological and nutritional interventions could be utilised to overcome iron-related impairments in a clinical setting. Speculatively, modalities developed to induce functional iron restriction via ferroportin inhibition (e.g., the hepcidin mimetic rusfertide, which can control iron flow to the erythron in polycythaemia vera [222]) may provide benefit for other disorders, perhaps in immunopathologies. Lastly, iron regulation may be more effective when in combination with other interventions. For instance, combining iron supplementation (intravenous or oral iron) with specific nutritional (e.g., vitamin A) or pharmacological (e.g., dopamine agonists) interventions identified via mechanistic investigation of iron deficiency in specific cell types could provide synergistic and efficacious benefits. Thus, the breadth of iron usage in cells offers not only challenges for understanding the effects of iron deficiency, but also opportunities to improve health.

Outstanding questions What tissues and cell types are most sensitive to iron deficiency in humans?

What is the hierarchy of sensitivity of cellular processes to iron deficiency and what factors (e.g., iron affinity, protein turnover, redundancy, iron trafficking, and localisation) drive this?

Given the role of iron in epigenetic processes, how does iron deficiency impact cell fate and function?

How does iron deficiency impact processes that are mediated by iron-dependent enzymes, including genome fidelity, bile acid synthesis, vitamin A and D metabolism, and steroidogenesis?

What molecular mechanisms underlie the many common clinical features associated with iron deficiency, and can they be targeted therapeutically?

As a comorbidity, how does iron deficiency exacerbate other diseases?

Can a mechanistic understanding of the role of iron in biology inform treatment of iron deficiency-associated disease?

Alt-text: Outstanding questions

## Glossary

**2-Oxoglutarate dependent dioxygenases (2-OGDDs)**: oxygen-, iron- and 2-oxoglutarate-requiring enzymes that mediate hydroxylation reactions.
**3-Hydroxyanthranilate 3,4-dioxygenase (HAAO)**: involved in kynurenine production.
**4-Hydroxyphenylpyruvate dioxygenase (HPD)**: involved in breakdown of tyrosine.
**5-aminolevulinic acid synthase (ALAS)-1/2**: enzymes involved in production of 5-aminolevulinic acid, the first step of haem synthesis.
**Alkylation B homolog (ALKBH) enzymes**: 2-OGDD enzymes involved in hydroxylation and demethylation of substrates, including DNA, mRNAs and tRNAs.
**Brain-derived neurotrophic factor (BDNF)**: growth factor that promotes neuronal survival and growth.
**Cytochrome P450 (CYP) enzymes**: haem-dependent enzymes, frequently mono-oxygenases, involved in cellular metabolism and xenobiotic detoxification.
**Deoxyhypusine monooxygenase (DOHH)**: conjugates a hypusine moiety to eIF5A.
**Electron transfer flavoprotein dehydrogenase (ETFDH)**: enzyme involved in linking β-oxidation to the ETC.
**Fatty acid desaturase (FADS)**: enzymes involved in formation of double bonds within fatty acid chains.
**Fe-S cluster assembly 1 and 2 (ISCA1/2)**: involved in production of [4Fe-4S] clusters.
**Glutaredoxins (GLRXs)**: proteins that utilise a glutathione cofactor to mediate redox reactions, including in Fe-S cluster synthesis.
**Histone lysine demethylases (KDMs)**: 2-OGDD enzymes involved in demethylation of lysine residues located in histone tails.
**Homogentisate 1,2-dioxygenase (HGD)**: involved in breakdown of tyrosine.
**Indoleamine 2,3-dioxygenase 1/2 (IDO1/2)**: involved in kynurenine production.
**Lipoic acid synthase (LIAS)**: protein-modifying enzyme that conjugates lipoic acid to proteins.
**Myeloperoxidase (MPO)**: haem-containing enzyme that uses H_2_O_2_ to oxidise its targets.
**NADH:ubiquinone oxidoreductase core subunit S1 (NDUFS1)**: core Fe-S cluster binding subunit of complex I.
**Phenylalanine hydroxylase (PAH)**: converts phenylalanine to tyrosine.
**Phosphoenolpyruvate carboxykinase (PCK)**: gluconeogenic enzyme that converts oxaloacetate to phosphoenolpyruvate.
**Phosphoribosyl pyrophosphate aminotransferase (PPAT)**: catalyses first step in *de novo purine synthesis.*
**Phytanoyl-CoA dioxygenase (PHYH)**: peroxisomal enzyme involved in phytanic acid breakdown.
**Polyunsaturated fatty acids (PUFAs)**: fatty acids containing multiple double bonds.
**Procollagen lysine 2-oxoglutarate 5-dioxygenase (PLOD) enzymes**: 2-OGDD enzymes that hydroxylate lysine residues during collagen synthesis.
**Solute carrier (SLC) proteins**: superfamily of membrane transport proteins mediating substrate transfer, including iron, glucose, and sodium.
**Stearoyl-CoA desaturase (SCD)**: converts unsaturated fatty acids to monosaturated fatty acids.
**Ten-eleven translocases (TETs)**: 2-OGDD enzymes involved in demethylation of DNA methyl groups.
**Thyroid peroxidase (TPO)**: involved in production of the thyroid hormones triiodothyronine (T3) and thyroxine (T4).
**Tryptophan 1,2-dioxygenase (TDO)**: involved in kynurenine production.
**Tryptophan hydroxylase 1/2 (TPH1/2)**: converts tryptophan to serotonin.
**Tyrosine hydroxylase (TH)**: involved in conversion of tyrosine to dopamine and noradrenaline.
**Ubiquinol-cytochrome c reductase, Rieske iron-sulfur polypeptide 1 (UQCRFS1)**: Fe-S cluster binding subunit of complex III.
**Xanthine dehydrogenase (XDH)**: a molybdenum and iron cofactor-containing enzyme that degrades xanthine in the purine degradation pathway.
